# Effectiveness and limitations of learning cardiopulmonary resuscitation with an automated external defibrillator in the curriculum of First Aid courses among lay people

**DOI:** 10.1186/cc10875

**Published:** 2012-03-20

**Authors:** U Kovačič, L Kosec

**Affiliations:** 1 Faculty of Medicine, University of Ljubljana, Slovenia; 2 General Hospital in Novo Mesto, Slovenia

## Introduction

The effectiveness and limitations of widespread promotion of cardiopulmonary resuscitation (CPR) with an automated external defibrillator (AED) among the laity was investigated. Early, qualitative and continuous cardiac massage has been stressed in the 2010 ERC guidelines. Since 2009 about 45,000 laypersons attended the mandatory First Aid courses for drivers (organised by Slovenian Red Cross), which include learning CPR with an AED.

## Methods

One hundred laypersons who attended 4-hour classes in CPR before the driving lessons were compared to 60 motivated laypersons who attended 6-hour classes in CPR before starting to work as lifeguards in pools. Sixty instructors served as the control group. All participants (randomly assigned in pairs) got the same 6-minute case-based scenario on a manikin. Rescuers were changing every 2 minutes. Basic skills in CPR were determined by the two instructors and by a sensored manikin. Massage was assessed as qualitative if at least 90% of massages were provided with proper hand placement, adequate compression depth and adequate frequency. We measured the response time from the call for help to the start of heart massaging and the percentage of the time of massaging regarding the total time from start of massaging to the end of the scenario.

## Results

Cardiac massage was not performed adequately in 48% of laypersons. This was statistically significantly more than among lifeguards (16%) and instructors (23%). The median response times of laypersons and lifeguards were 15 seconds and 16 seconds, respectively; this was statistically (*P *< 0.05) longer than instructors (12 seconds). The median percentage of the time of massaging in group of laypersons was 51% (56 to 58%, 25th to 75th percentiles), which was statistically significantly smaller than in the group of lifeguards (64%, 62 to 66%) and in the control group (67%, 62 to 69%).

## Conclusion

The majority of all laypersons approach CPR in about 15 seconds from identification of unconsciousness. However, only about one-half of laypersons after the mandatory CPR course perform qualitative cardiac massage, which is significantly less than among motivated laypersons. The latter perform qualitative massage and achieve the same percentage of the massaging time as instructors. Results suggest that widespread promotion of the CPR protocol with an AED among laypersons has limitations. Therefore, education of laypersons should particularly focus on groups that have intrinsic motivation.

